# Evaluation of potential changes in liver and lung tissue of rats in an ischemia-reperfusion injury model (modified pringle maneuver)

**DOI:** 10.1371/journal.pone.0178665

**Published:** 2017-06-12

**Authors:** Silvio Henrique Freitas, Renata G. S. Dória, Rachel S. Bueno, William B. Rocha, Jair R. E. Filho, Julieta R. E. Moraes, Atanásio Serafin Vidane, Carlos E. Ambrósio

**Affiliations:** 1 Faculty of Veterinary Medicine, University of Cuiaba, Cuiabá, Mato Grosso, Brazil; 2 Department of Veterinary Medicine, Faculty of Animal Sciences and Food Engineering, University of São Paulo, Pirassununga, São Paulo, Brazil; 3 Department of Basic Sciences, Faculty of Animal Sciences and Food Engineering, University of São Paulo, Pirassununga, São Paulo, Brazil; 4 Faculty of Veterinary Medicine, University of Cuiaba, Cuiabá, Mato Grosso, Brazil; 5 Graduate Program in Animal Science, School of Agricultural Sciences and Veterinary Medicine - Pontifícia Universidade Católica do Paraná (PUCPR), São José dos Pinhais, Paraná, Brazil; 6 Department of Pathology, Faculty of Agriculture and Veterinary Sciences, São Paulo State University Júlio de Mesquita Filho, Jaboticabal, São Paulo, Brazil; 7 Veterinary Faculty, Eduardo Mondlane University, Maputo, Mozambique; University of Colorado Denver, UNITED STATES

## Abstract

In surgical procedures involving the liver, such as transplantation, resection, and trauma, a temporary occlusion of hepatic vessels may be required. This study was designed to analyze the lesions promoted by ischemia and reperfusion injury of the hepatic pedicle, in the liver and lung, using histopathological and immunohistochemical techniques. In total, 39 Wistar rats were divided into four groups: control group (C n = 3) and ischemia groups subjected to 10, 20, and 30 minutes of hepatic pedicle clamping (I10, n = 12; I20, n = 12; I30, n = 12). Each ischemia group was subdivided into four subgroups of reperfusion (R15, n = 3; R30, n = 3; R60, n = 3; R120, n = 3), after 15, 30, 60, and 120 minutes of reperfusion, respectively. Significant differences were observed in the liver parenchyma (P < 0.05) between the values of microvesicles and hydropic degeneration at different times of ischemia and reperfusion. However, the values of vascular congestion, necrosis, and pyknotic nuclei showed no significant differences (P > 0.05). In the lung parenchyma, a significant difference was observed (P < 0.05) between the values of alveolar septal wall thickening and inflammatory infiltration at different times of ischemia and reperfusion. However, there was no significant difference (P < 0.05) between the values of vascular congestion, bronchial epithelial degeneration, interstitial edema, and hemorrhage. The positive immunoreactivity of caspase-3 protein in the liver parenchyma (indication of ongoing apoptosis), showed no significant differences (P > 0.05) at different times of ischemia and reperfusion. In the pulmonary parenchyma, the immunoreactivity was not specific, and was not quantified. This study demonstrated that the longer the duration of ischemia and reperfusion, the greater are the morphological lesions found in the hepatic and pulmonary parenchyma.

## Introduction

In liver lesions and transplants, temporary blockage of hepatic blood flow is sometimes necessary to facilitate surgical maneuvers [1,2]. Since 1908, Pringle proposed full clamping of the hepatic pedicle (hepatic artery, portal vein, and the common bile duct) to restrain hepatic hemorrhages [3]. This procedure results in splenic congestion and consequently, poor blood perfusion in the liver, stomach, small intestine, anterior part of the large intestine, pancreas, and spleen [4–8]. Upon restoring blood flow to the ischemic region, the injuries caused by ischemia might be aggravated further [9–12].

The ischemic event begins with an inflammatory response perpetuated after blood reperfusion due to production of reactive oxygen species (ROS). This phenomenon is known as ischemia-reperfusion injury and involves complex pathophysiological mechanisms and metabolic pathways, which are not well understood; however, the end result is organ failure and patient death [8–10].

Apoptosis is an important biological process that controls many vital processes and could be associated with conditions involving the enhancement of ROS [13]. In cell death after ischemia and reperfusion, necrosis and apoptosis coexist. The proposed mechanisms include ROS formation during reperfusion in the parenchymal cells, endothelial cells, and lymphocytes recruited to the injury site. They cause cell damage directly through a variety of secreted molecules and indirectly by promoting the synthesis of pro-inflammatory mediators [14].

After necrosis, the cell membrane integrity is lost and the intracellular content is typically released, thus causing inflammation in the surrounding tissue. The morphology of necrotic cells is characterized by increased eosinophilia, vacuoles in the cytoplasm after digestion of cytoplasmic organelles, and nuclear changes encompassing karyolysis, cariorrexis, and pyknosis. In apoptosis, the plasma membrane remains intact and the cell structure is modified, and so, these cells are rapidly phagocytosed without releasing their contents or triggering an inflammatory reaction. The morphological features of apoptosis include reduction in cell size, chromatin condensation at the periphery, and formation of apoptotic bodies and cytoplasmic bubbles. The specific biochemical changes are characterized by protein degradation involving the activation of cysteine proteases called caspases and DNA decomposition [15,16]. Based on these peculiarities, apoptosis can be characterized using immunohistochemical techniques to evaluate the expression pattern of cleaved caspase-3 [17,18]. Programmed cell death markers such as cysteine protease 32, known as caspase-3 (proteolytic enzyme), have been studied using paraffin blocks derived from different tissues, including the liver, to quantify their expression [17].

Thus, this study was designed to evaluate potential histopathological changes and to immunohistochemically characterize apoptosis in the liver and lung tissues of rats following an ischemia-reperfusion injury, using a model (modified Pringle maneuver) that simulates clinical situations such as gastric dilatation-volvulus, nephrosplenic or gastrosplenic entrapment, spleen torsion, and intestinal intussusception, which involve compromised blood flow in the hepatic pedicle and subsequently, organs associated with the portal system [19–25].

## Materials and methods

All techniques and procedures performed in this study were approved by the University of Cuiabá ethical committee for animal research (no. 2009–097).

In this study, we used 39 male Wistar rats (*Rattus norvegicus albinus*) three months old, with an average weight of 300 grams. The animals were housed in cages at room temperature maintained by air conditioning, 65% humidity, and brightness controlled artificially by fluorescent lamps (daylight—Phillips—40 Watts), with 12 hours of light and 12 hours of darkness. The animals were randomly distributed to four experimental groups: Control group (C, n = 3), animals with no clamped hepatic pedicle; Ischemia group 10 (I10, n = 12) hepatic pedicle subjected to 10 minutes clamping; Ischemia group 20 (I20, n = 12) hepatic pedicle subjected to 20 minutes clamping, and Ischemia group 30 (I30, n = 12) hepatic pedicle subjected to 30 minutes clamping. Each ischemia group was further divided into four subgroups: Subgroup 15 (R15, n = 3) subjected to 15 minutes of reperfusion after hepatic pedicle declamping; Subgroup 30 (R30, n = 3) subjected to 30 minutes of reperfusion after hepatic pedicle declamping; Subgroup 60 (R60, n = 3) subjected to 60 minutes of reperfusion after hepatic pedicle declamping, and Subgroup 120 (R120, n = 3) submitted to 120 minutes of reperfusion after hepatic pedicle declamping.

The animals were anesthetized with a mixture of xylazine (25 mg/kg) and ketamine (50 mg/kg), via intraperitoneal administration. After abdominal trichotomy and antisepsis with povidone-iodine-alcohol, pre-umbilical median celiotomy was performed, the hepatic pedicle was accessed, and subjected to the clamping and unclamping procedure.

The animals of control group (C) were only submitted to pre-umbilical median celiotomy without clamping the hepatic pedicle. After median celiotomy, the hepatic pedicles of the I10 group animals were clamped for 10 minutes using vascular clamps (Bulldog). After this period, the R15, R30, R60, and R120 subgroups were subjected to hepatic pedicle declamping and reperfusion for 15, 30, 60, and 120 minutes, respectively. The I20 and I30 groups received similar treatment as the I10 group, with the period of hepatic pedicle clamping extended to 20 and 30 minutes respectively.

At the end of the period established for each ischemia and reperfusion, the anesthetized animals were euthanized with intravenous sodium thiopental followed by intravenous potassium chloride, drugs that promoted cardiorespiratory arrest and death. The liver and lung were then collected and immersed in 10% formaldehyde phosphate buffer solution for 24 hours. These materials were processed for histopathological and immunohistochemical assays.

For histopathological assays, the liver and lung tissues were embedded in paraffin, sliced into 5-μm thick sections and stained with routine hematoxylin-eosin. The slides were observed by light microscopy (Axiolab 2.0 Carl Zeiss), using the 40x objective. The histological assessments were performed by an independent blind observer.

The quantitative method was used to analyze the presence and intensity of the histopathological lesions found in the liver and lung tissues. Therefore, 10 random fields (400x) were observed in each section and the lesions were classified as absent (0) to ++++, according to their intensity, and then converted to numbers for statistical analysis: absence = 0, +/- = 0.5, + = 1, ++ = 2, +++ = 3, and ++++ = 4 [26].

In the liver, the following features were analyzed: vascular congestion—increase of capillary caliber; microvesicles—the presence of small circular vesicles containing lipids in the cytoplasm; hydropic degeneration—increase in cell volume by water influx into the cell, showing granular cytoplasm, large nuclei, and sparse chromatin; necrosis—characterized by nuclear condensation or absence of nuclei, intense cytoplasmic eosinophilia and destruction, loss of hepatocyte cord architecture (the loss of hepatocellular trabeculae), and pyknotic nuclei—characterized by nuclear condensation and intense staining. In the lung, we analyzed the occurrence of: vascular congestion—presence of dilated vessels in the lung stroma; degeneration of bronchial epithelium—presence of necrotic cells; thickening of the alveolar septum—increased thickness of the septa; interstitial edema—presence of a weak and homogeneous eosinophilic substance in the interstitial space; hemorrhage—presence of red blood cells in the alveolar interstitium, and inflammation—presence of leukocytes, particularly neutrophils distributed in several parts of the lung.

For immunohistochemical assays, 5 μm tissue sections were deparaffinized by serial immersion in xylene and alcohol and the antigenic sites were recovered in fluent vapor for 30 minutes. The slides were then washed twice with 0.1M Tris saline, pH 7.5 (TBS) for 5 minutes in a shaker followed by blocking of endogenous peroxidase by immersion in 1% hydrogen peroxide for 30 minutes at room temperature. The slides were then washed again in TBS (2x, 5 minutes), and to block non-specific staining, the samples were incubated with normal donkey serum diluted in 0.2 M phosphate buffer solution, pH 7.4 (PBS). The samples were labeled overnight with primary antibodies (1:200) (Rheabiotech Ltd., Campinas, Brazil), at 4°C, in the dark and then, repeatedly washed in TBS containing 0.1% Tween 20. The sections were then incubated for 30 minutes at room temperature with anti-rabbit IgG secondary antibody conjugated with biotin (1:400) (LSAB2 kit, Dako—Agilent Technologies, Germany). Subsequently, the samples were incubated with AB reagent (avidin-biotin complex) for 30 minutes at room temperature (LSAB2 kit, Dako—Agilent Technologies, Germany) and then with DAB (Sigma Chemical Co., St. Louis, USA) for 5 minutes at room temperature. The sections were then counterstained with hematoxylin. The brown stain represented by caspase-3 expression was evaluated using an optical microscope (400x) (Carl Zeiss, model Scope.A1). To analyze the relative amount of cellular profiles per unit area we used the AxioVision 4.8—Carl Zeiss software. For this, 10 random fields of each section were observed and the mean percentage of immunostained area in relation to the total field area was calculated.

A Kruskal-Wallis nonparametric statistical test was performed to verify the possible difference between the periods of ischemia-reperfusion. When a difference in the groups was observed, the Dunn multiple comparison test was performed to determine the pairs of groups that differed. The data were analyzed using the SAS program (SAS, version 9.2) at a significance level of 5%.

## Results

**Fig 1:**
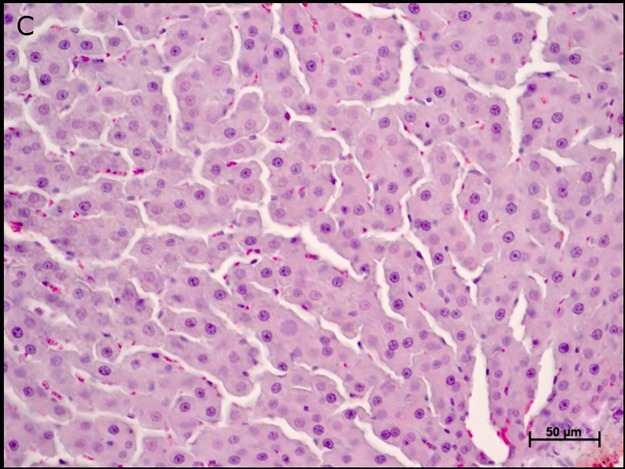
Photomicrographs of the liver parenchyma of control group animals (C). Note: central vein, hepatocytes, and sinusoidal capillaries. Hematoxylin-eosin staining (HE).

The hepatic parenchyma of the control group animals (C) was preserved: central vein, hepatocytes, and sinusoidal capillaries (Fig 1).

**Fig 2:**
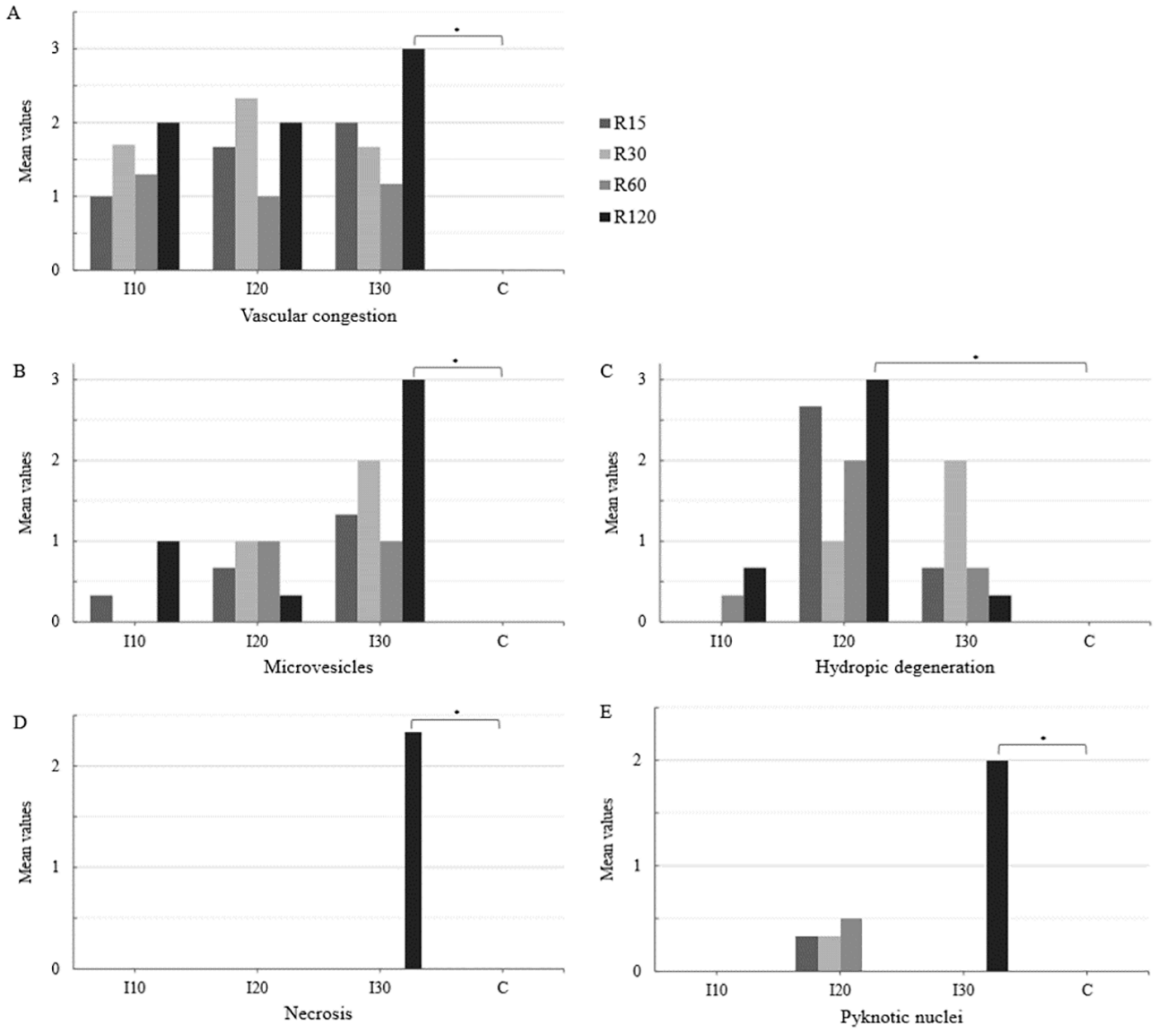
Mean values of vascular congestion (A), microvesicles (B), hydropic degeneration (C), necrosis (D), and pyknotic nuclei (E) in the liver parenchyma of the ischemia (I), reperfusion (R), and control (C) group animals. The data were recorded by optical microscopy after hematoxylin-eosin staining (HE). Ischemic groups: I10–10 minutes of ischemia, I20–20 minutes of ischemia and I30–30 minutes of ischemia. Reperfusion subgroups: R15–15 minutes of reperfusion, R30–30 minutes of reperfusion, R60–60 minutes of reperfusion and R120–120 minutes of reperfusion (*P < 0.05).

**Fig 3:**
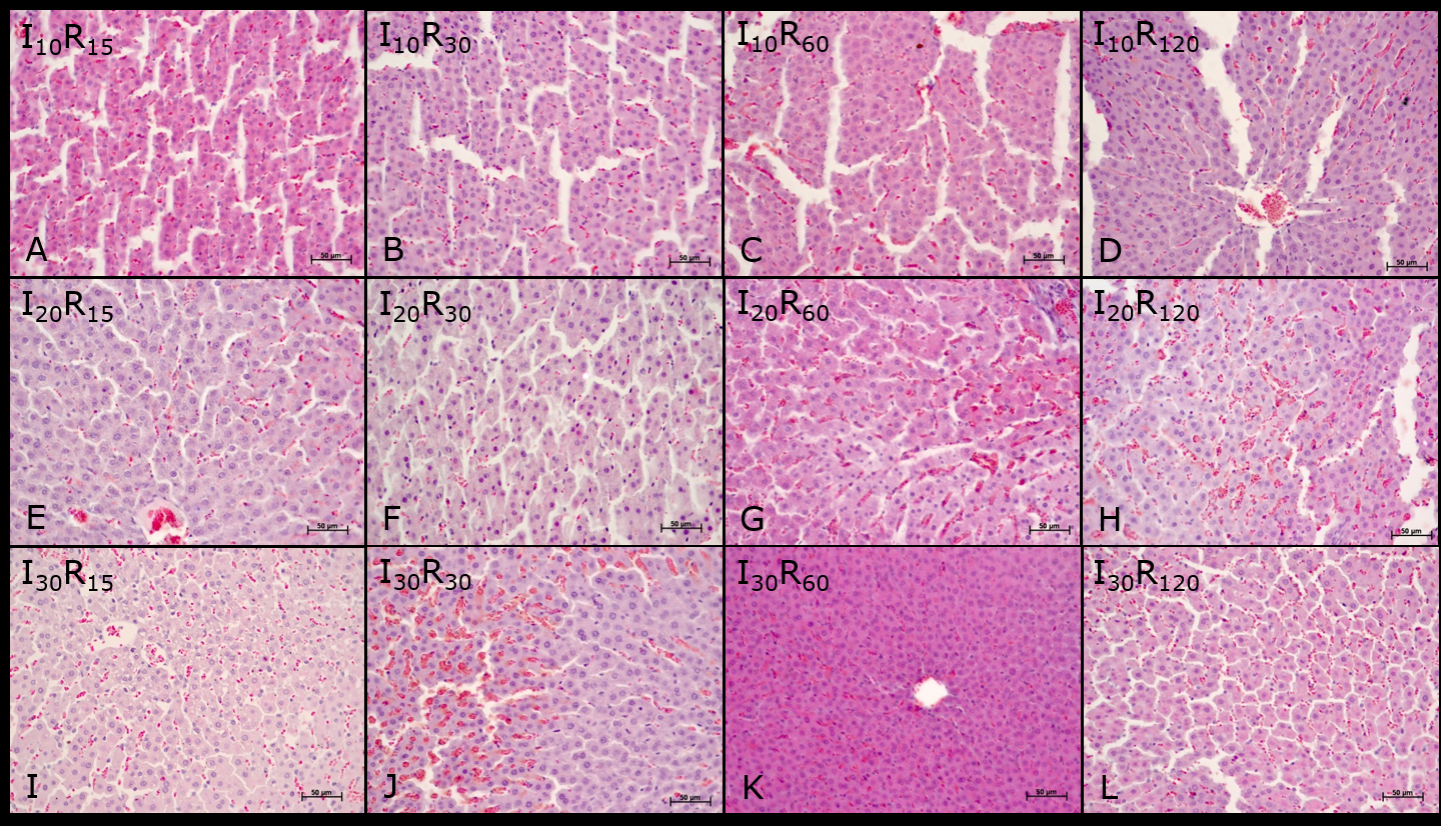
Liver parenchyma photomicrographs of Wistar rats subjected to ischemia and reperfusion. Groups (10, 20, and 30 minutes ischemia) and subgroups (15, 30, 60, and 120 reperfusion): I10 and R15 (A), I10 and R30 (B), I10 and R60 (C), I10 and R120 (D), I20 and R15 (E), I20 and R30 (F), I20 and R60 (G), I20 and R120 (H), I30 and R15 (I), I30 and R30 (J), I30 and R60 (K) e I30 and R120 (L). Note: vascular congestion, microvesicles, hydropic degeneration, necrosis, and pyknotic nuclei. Hematoxylin-eosin staining (HE).

In the ischemia groups and reperfusion subgroups (I and R), a significant difference in the mean values of microvesicles (I30> I20> I10> C) and hydropic degeneration (I20> I30> I10> C) was observed at different times of ischemia. Therefore, the mean values of microvesicles (I30 and R120> C) and hydropic degeneration (I20 and R120> C) were statistically different from those of group C (Figs 2 and 3).

No significant differences were found in the mean values of vascular congestion, necrosis, and pyknotic nuclei at different times of ischemia. The mean values of vascular congestion, necrosis and pyknotic nuclei in the I30 and R120 group, differed from those of the C group (I30 and R120> C), see Figs 2 and 3.

**Fig 4:**
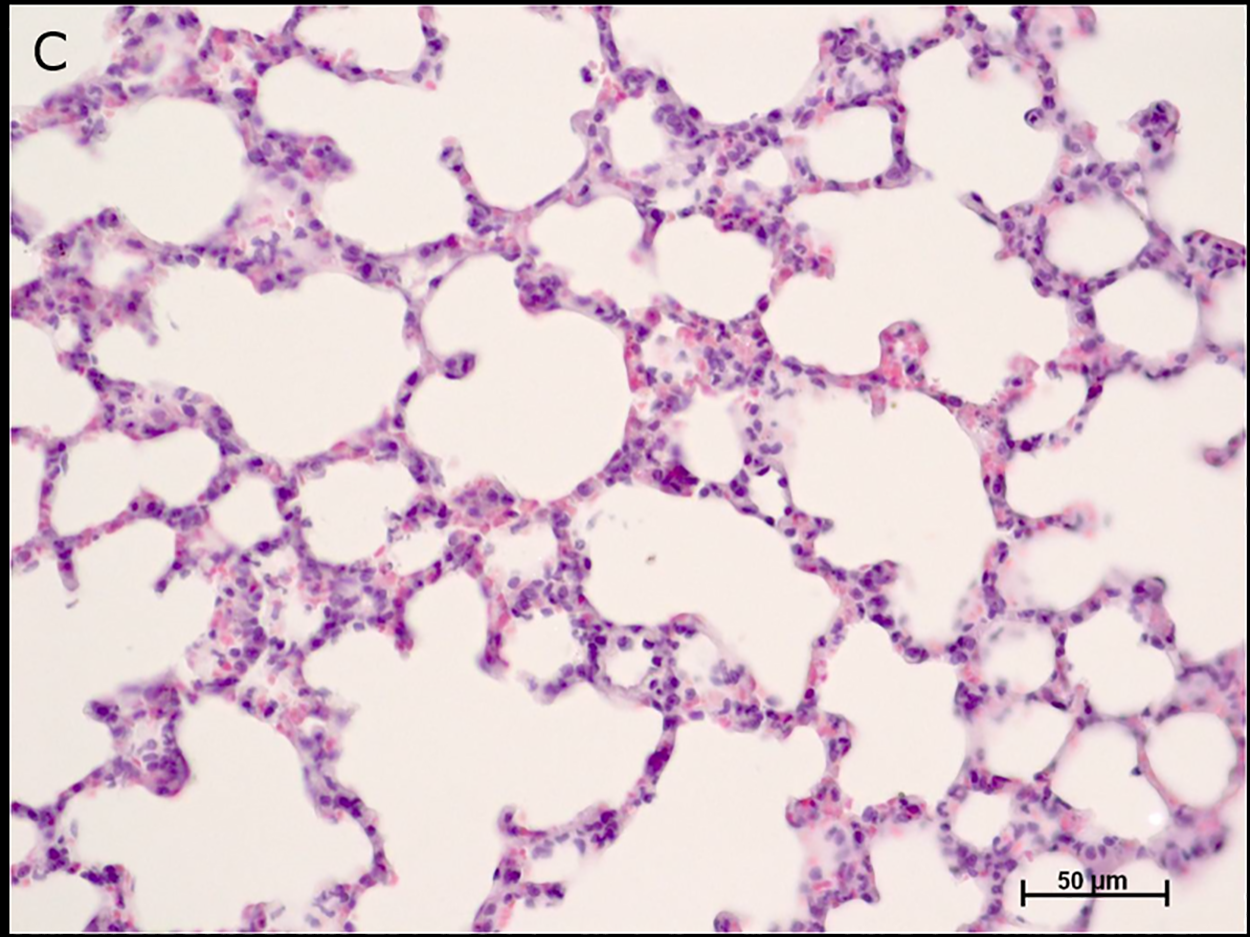
Photomicrographs of the pulmonary parenchyma of control group animals (C). Note: alveolar septum, alveolar duct, alveolar sac, alveoli, and bronchioles. Hematoxylin-eosin staining (HE).

The pulmonary parenchyma of control group animals (C) showed no changes in the alveolar septum, alveolar duct, alveolar sac, alveoli, and bronchioles (Fig 4).

**Fig 5:**
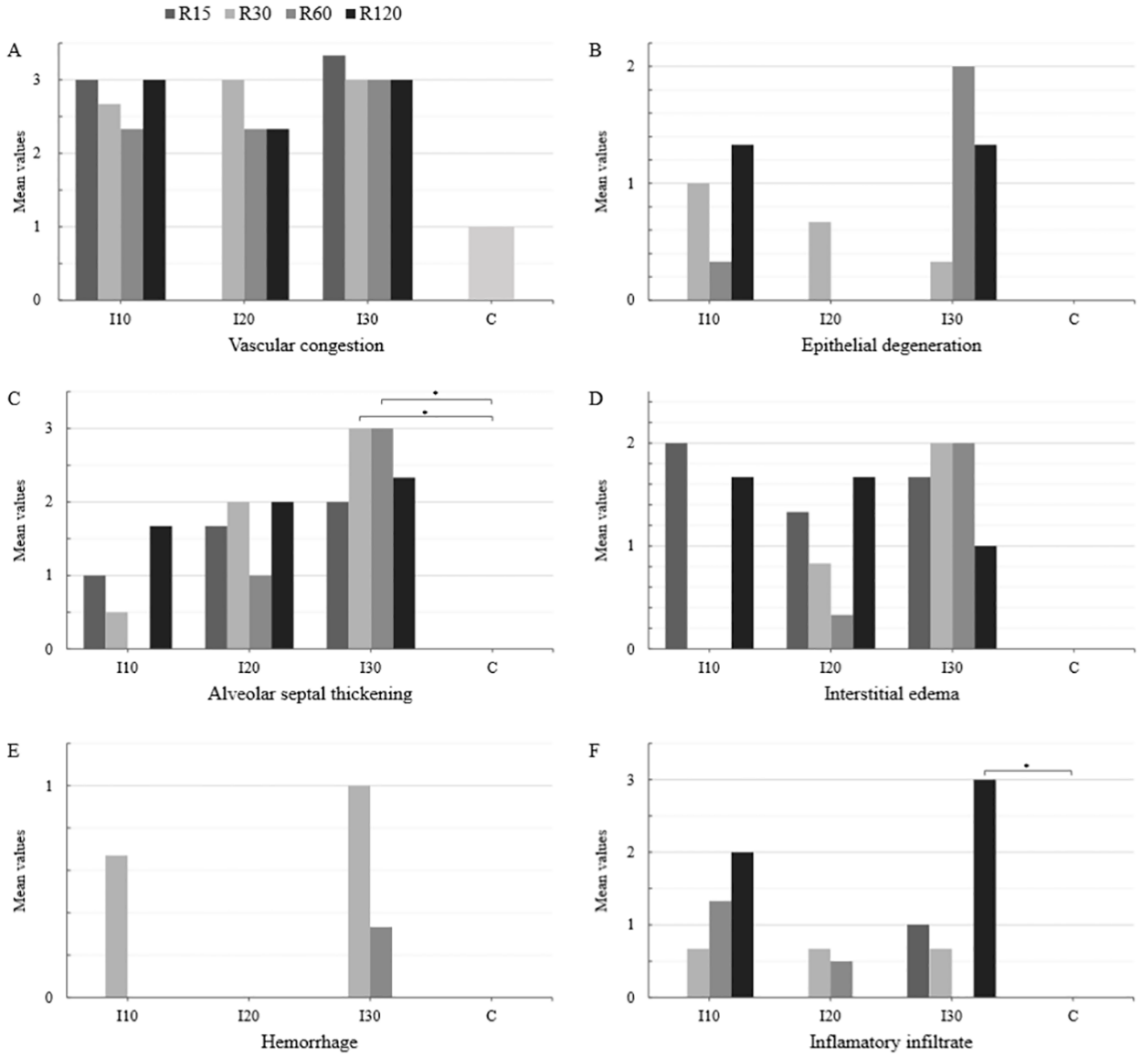
Mean values of vascular congestion (A), degeneration of bronchial epithelium (B), alveolar septal thickening (C), interstitial edema (D), hemorrhage (E), and inflammatory infiltrate (F) in the pulmonary parenchyma of the ischemia (I), reperfusion (R), and control (C) group animals. The data were recorded by optical microscopy after hematoxylin-eosin staining (HE). Ischemic groups: I10 –ischemia 10 minutes, I20 –ischemia 20 minutes and I30 –ischemia 30 minutes. Reperfusion subgroups: R15 –reperfusion 15 minutes, R30 –reperfusion 30 minutes, R60 –reperfusion 60 minutes, and R120 –reperfusion 120 minutes. (*P < 0.05).

**Fig 6:**
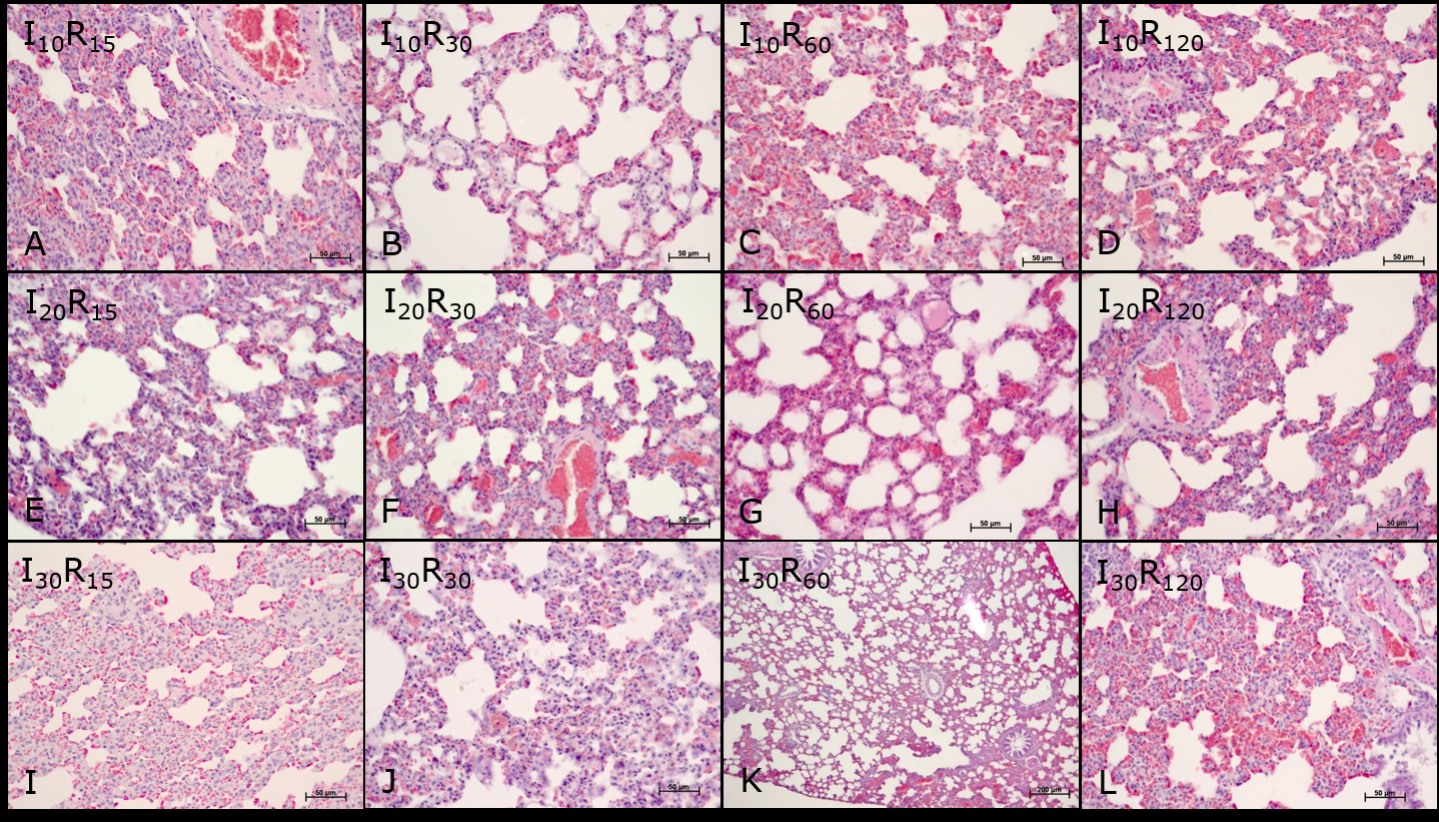
Photomicrographs of the pulmonary parenchyma of Wistar rats subjected to ischemia and reperfusion. Groups (10, 20, and 30 minutes ischemia) and subgroups (15, 30, 60, and 120 reperfusion): I10 and R15 (A), I10 and R30 (B), I10 and R60 (C), I10 and R120 (D), I20 and R15 (E), I20 and R30 (F), I20 and R60 (G), I20 and R120 (H), I30 and R15 (I), I30 and R30 (J), I30 and R60 (K), I30 and R120 (L). Note: vascular congestion, degeneration of bronchial epithelium, alveolar septal thickening, interstitial edema, and hemorrhage. Hematoxylin-eosin staining (HE).

In the ischemia groups and reperfusion subgroups (I and R), a significant difference in the mean values of alveolar septal thickening (I30> I20> I10> C) and inflammatory infiltrate (I30> I20> I10> C) was observed at different times of ischemia. Therefore, the mean values of alveolar septal thickening (I30 and R30 = I30 and R60 > C) and inflammatory infiltrate (I30 and R120 > C) were statistically different from those of group C (Figs 5 and 6).

No significant differences were found in the mean values of vascular congestion, degeneration of the bronchial epithelium, interstitial edema, and hemorrhage at different times of ischemia and reperfusion when compared with those of control group animals C (Figs 5 and 6).

**Fig 7:**
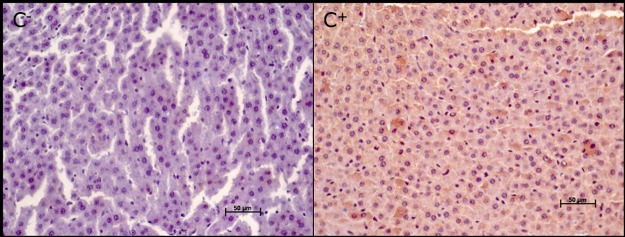
Photomicrographs of the hepatic parenchyma in control group animals: Negative control (C-) and positive control (C+). Note the positive immunoreactivity for caspase-3 in the C+ group (brown coloration).

The caspase-3 protein evaluated by immunohistochemistry was not expressed in the liver parenchyma of the negative control group (C^-^) but was expressed in the positive group (C^+^) (Fig 7).

**Fig 8:**
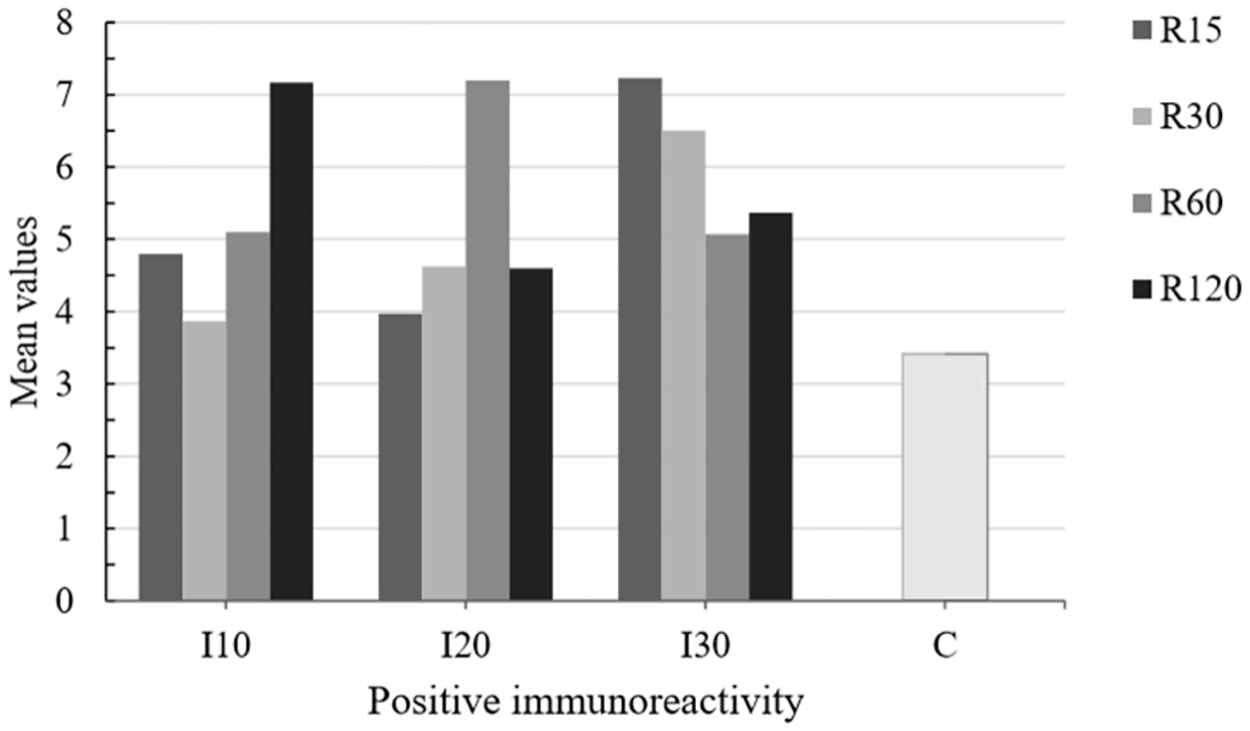
Mean values of positive immunoreactivity for caspase-3 protein in the hepatic parenchyma of ischemia (I), reperfusion (R) and control (C) group animals. The data were recorded by optical microscopy after immunohistochemical staining. Ischemic groups: I10 –ischemia 10 minutes, I20 –ischemia 20 minutes, and I30 –ischemia 30 minutes. Reperfusion subgroups: R15 –reperfusion 15 minutes, R30 –reperfusion 30 minutes, R60 –reperfusion 60 minutes, and R120 –reperfusion 120 minutes. (P < 0.05).

**Fig 9:**
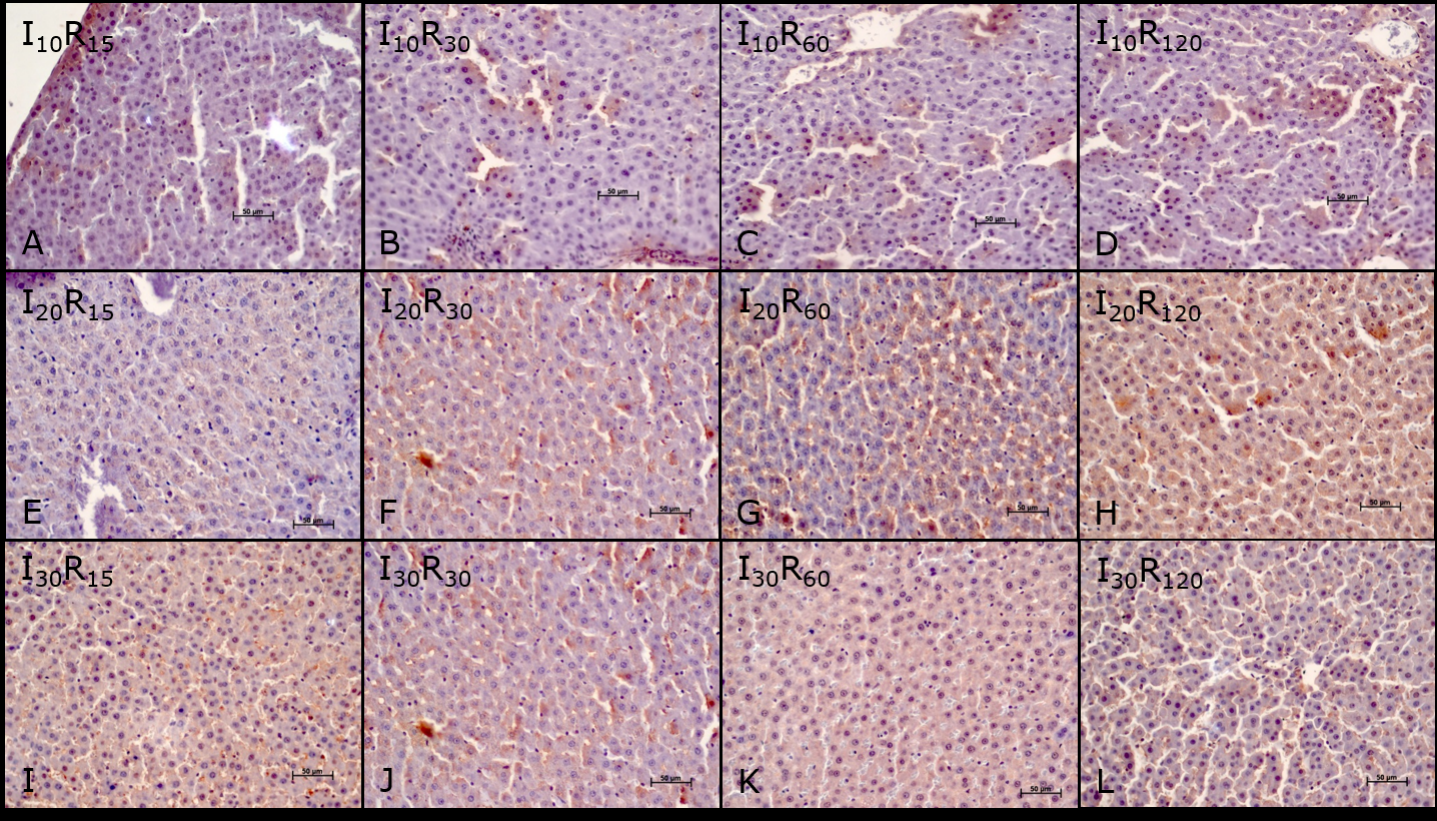
Photomicrographs of the hepatic parenchyma of Wistar rats subjected to ischemia and reperfusion. Groups (10, 20, and 30 minutes ischemia) and subgroups (15, 30, 60 and 120 minutes reperfusion): I10 and R15 (A), I10 and R30 (B), I10 and R60 (C), I10 and R120 (D), I20 and R15 (E), I20 and R30 (F), I20 and R60 (G), I20 and R120 (H), I30 and R15 (I), I30 and R30 (J), I30 and R60 (K) e I30 and R120 (L). Note the positive immunoreactivity for caspase-3 protein (brown coloration).

A positive immunoreactivity for caspase-3 protein was also observed in the hepatic parenchyma of all experimental group animals: C^+^ group and I10, I20, and I30 (R15, R30, R60, R120) groups (Figs 8 and 9).

In the ischemia and reperfusion subgroups (I and R), no significant difference were found in the mean values of positive immunoreactivity for caspase-3 at different times of ischemia (Figs 8 and 9).

The pattern of positive immunoreactivity for caspase-3 protein in lung tissue did not allow quantification as the brown coloration was present in the entire tissue parenchyma.

## Discussion

The period of ischemia used in this study was based on previous studies that evaluated the intensity of lesions induced in the liver and remote organs [4,5,7,27–34]. According to literature, the recommended period of hepatic ischemia varies from 30 to 100 minutes [4,5,28,29,33], with the greatest periods related to actions involving partial hepatic ischemia with vascular shunts, and shorter periods related to actions involving complete ischemia without vascular shunts as described by Sébe et al, (2000) [4] and Camargo et al, (2003) [5].

In this study, a comparison of ischemia times ranging from 10 to 30 minutes, with progressive periods of reperfusion, showed increasing changes in tissue features, suggesting a time-dependent relation between ischemia/reperfusion and the severity of liver and lung injury.

Ischemia is defined as the absence of blood flow resulting in reduction of oxygen and substrate supply to tissues, with consequent accumulation of deleterious metabolites in the cells [4,20]. In humans, if the blood flow is not restored, acute ischemia can cause various degrees of damage ranging from minor necrosis to total commitment of vital organs. When blood flow is restored after acute and mild ischemia, there is usually no morpho-functional impairment of the involved organs. However, severe ischemia can lead to a post-revascularization syndrome complex, which often results in failure and loss of vital organs [20]. No death occurred in this study, probably because the maximum time for evaluation after ischemia (I30) was 120 minutes of reperfusion (R120), and at the end of each time proposed, the animals were euthanized for sample collection.

The variables analyzed in the liver parenchyma (vascular congestion, microvesicles, hydropic degeneration, necrosis, and pyknotic nuclei) and in the pulmonary parenchyma (vascular congestion, degeneration of the bronchial epithelium, thickening of alveolar septa, interstitial edema, red blood cells, and inflammatory infiltrate), although not part of surgical profile tests (surgical risk) for the majority of patients subjected to liver surgery, must be considered in cases where total or temporary ischemia followed by reperfusion is performed.

In this study, after total cross-clamping of the hepatic pedicle (I10, I20, I30) and reperfusion (R15, R30, R60, R120), it was observed that longer duration of ischemia and reperfusion resulted in greater vascular congestion, microvesicles, and hydropic degeneration in the liver parenchyma. Hepatic parenchyma necrosis was only observed after 30 minutes of ischemia and 120 minutes of reperfusion (I30 and R120). Pyknotic nuclei were not observed after 10 minutes of ischemia (I10), however, after 20 minutes of ischemia (I20) we found that a longer reperfusion period resulted in greater appearance of pyknotic nuclei. Therefore, after 30 minutes of ischemia, pyknotic nuclei were only present after 120 minutes of reperfusion (I30 and R120). Nevertheless, the mean values between the ischemia times were significantly differences only for microvesicles and hydropic degeneration.

In the pulmonary parenchyma, vascular congestion was observed after 10, 20, and 30 minutes of ischemia, with no significant differences in relation to the time of reperfusion and between different times of ischemia. Degeneration of the bronchial epithelium and interstitial edema were observed in the lung parenchyma after 10, 20, and 30 minutes of ischemia. It was observed that, the longer the duration of ischemia and reperfusion, the greater was the thickening of the alveolar septa. Hemorrhage was present in the lung parenchyma after 10 minutes of ischemia and 30 minutes of reperfusion, and also after 30 minutes of ischemia followed by 60 minutes of reperfusion. Inflammatory infiltrate was found in the pulmonary parenchyma after 10, 20, and 30 minutes of ischemia, and was greater when the time to reperfusion was longer. Besides the variations between the different times of ischemia (I10, I20, I30) and reperfusion (R15, R30, R60, R120), the mean values between the ischemic times were only significantly differences for the thickening of alveolar septa and inflammatory infiltrate.

Oxygen-derived free radicals are highly reactive molecules capable of inducing cell and DNA damage. Hydrogen peroxide (OH-) is not a free radical, but when produced in abundance in the presence of metal catalysts (i.e. iron), it forms reactive hydroxyl radicals, which can lead to local or distant cell damage through the Haber-Weiss reaction [9,10,26,35,36,37]. In this study, we demonstrated the local significant changes in the liver characterized by the presence of microvesicles (I30 and R120) and hydropic degeneration (I20 and R120). However, the mean values for vascular congestion, necrosis, and pyknotic nuclei were not statistically different from those of the control group. Regarding the distant lesions in the lung, the mean values of vascular congestion, degeneration of the bronchial epithelium, interstitial edema, and pulmonary hemorrhage were not significantly different between the groups of ischemia and reperfusion. The mean values of alveolar septal thickening and inflammatory filtrate showed significant differences between the groups of ischemia and reperfusion. The ischemia and reperfusion periods in this study probably induced only the activation of inflammatory mechanisms and the production of free radicals, which were not enough to cause liver and lung failure.

Using immunohistochemistry, we demonstrated that the longer the duration of ischemia (I10, I20, I30) and reperfusion (R15, R30, R60, R120), the greater was the expression of positive immunoreactivity for caspase-3 protein in the liver parenchyma. These findings indicate enhanced apoptosis or programmed cell death, even though the mean values were not significantly different. The exact mechanism of cell death in ischemia and reperfusion injury is not yet well understood. A previous study [38] showed that, after selective liver ischemia for 60 minutes, cell death in the early phase of injury was mainly caused by necrosis, whereas in the late phase (24 hours of reperfusion), cell death was caused by apoptosis. A time-dependent increase in the number of apoptotic cells was also observed in the liver in relation to ischemia [38,39]. Kohli et al. (1999) [39] reported that 40–60% of hepatocytes undergo apoptosis during reperfusion. This finding suggests that inhibition of apoptosis plays a key role in reducing ischemia-reperfusion lesions in the liver, encouraging further studies aiming to develop modulation strategies. However, in this study, the mean values of positive immunoreactivity for caspase-3 protein in the hepatic parenchyma showing ongoing apoptosis, presented no statistically significant differences between the different periods of ischemia and reperfusion.

## Conclusion

This study demonstrated that, the longer the duration of ischemia and reperfusion, the greater are the morphological lesions found in the hepatic and pulmonary parenchyma. There is no difference in the expression values of positive immunoreactivity for caspase-3 protein present in hepatic parenchyma at different periods of ischemia and reperfusion.

## References

[1] Silva OC Jr, Centurion S, Pacheco EG, Brisotti JL, Oliveira AF, Dal Sasso K. Basics aspects of the ischemia reperfusion injury and of the ischemic preconditioning. Acta Cir Bras. 2002;17: 96–100.

[2] Araújo FA Jr, Braz MN, Rocha Neto OG, Costa FD, Brito MVH. Copaiba oil effect in rat aminotransferases submitted to hepatic ischemic and reperfusion with and without preconditioning. Acta Cir Bras. 2005;20: 93–99. 15810470 10.1590/s0102-86502005000100013

[3] Pringle JH. Notes on the arrest of hepatic hemorrhage due to trauma. Ann Surg. 1908;48: 541–549. 17862242 10.1097/00000658-190810000-00005 PMC1406963

[4] Sébe AA, Nigro AJT, Gomes PO, Simões MJ. Effects of clamping of the hepatic pedicle in the intestines. Acta Cir Bras. 2000;15: 4–8.

[5] Camargo LM, Evêncio Neto J, Freitas SH, Simões MJ, Gomes PO, Sébe AA. Ultrastructural aspects of the intestinal villi after hepatic pedicle clamping in rats. Acta Cir Bras. 2003;18: 509–513.

[6] Brasileiro JL, Fagundes DJ, Miiji LON, Oshima CTF, Teruya R, Marks J, et al Ischemia and reperfusion of the soleus muscle of rats with pentoxifylline. J Vas Bras. 2007;6: 50–63.

[7] Freitas SH, Evencio Neto J, Doria RGS, Mendonca FS, Simões MJ, Camargo LM, et al Macroscopic, morphologic, and morphometric aspects of the spleen of rats after total clamping of the hepatic pedicle. Ciênc Anim Bras. 2009;10: 1218–1226.

[8] Ferraz AAB, Campos JM, Evangelista LF, Coelho ARB, Araújo-Filho JG, Ferraz EM. Myoelectric activity of the small bowel of dogs submitted to partial occlusion of the portal vein. Arq Bras Cir Digest. 2011;24: 20–25.

[9] Miranda LEC, Viaro F, Caneviva R, Evora PRB. The experimental basis of hepatic ischemia-reperfusion injury: review. Acta Cir Bras. 2004;19: 1–10.

[10] Silva FN. Refinetti RA, Eulálio JMR. Biochemical assessment of ischemic preconditioning after hepatic ischemia and reperfusion in rats. Rev Col Bras Cir. 2006;33: 393–397.

[11] Dello SAWG, Reisinger KW, van Dam RM, Bemelmans MHA, van Kuppevelt TH, van den Broek MA, et al Total intermittent pringle maneuver during liver resection can induce intestinal epithelial cell damage and endotoxemia. PLoS One. 2012;7: 1–6 e30539. 10.1371/journal.pone.0030539 22291982 PMC3265485

[12] Nomi T, Fuks D, Agrawal A, Govindasamy M, Araki K, Gayet B. Modified Pringle maneuver for laparoscopic liver resection. Ann Surg Oncol. 2015;22: 852 10.1245/s10434-014-4088-5 25223928

[13] Grivicich I, Regner A, Rocha AB. Apoptosis: Programmed Cell Death. Rev Bras Cancerol. 2007;53: 335–343.

[14] Daemen MRC, Vries B, Buurman WA. Apoptosis and inflammation in renal reperfusion injury. Transplantation. 2002;73: 1693–1700. 12084988 10.1097/00007890-200206150-00001

[15] Toledo-Pereyra LH, Suzuki S. Neutrophils, cytokines and adhesion molecules in hepatic ischemia and reperfusion injury. J Am Coll of Surg. 1994;179: 758–762. 7952491

[16] Lopez-Neblina F, Toledo AH, Toledo-Pereyra LH. Molecular biology of apoptosis in ischemia and reperfusion. J of Invest Surg. 2005;18: 335–350. 16319055 10.1080/08941930500328862

[17] Anwar S, Ambros RA, Jennings TA, Ross J, Beza A, Mian B, et al Expression of cysteine protease protein 32 in prostatic adenocarcinoma correlates with tumor grade. Arch Pathol Lab Med. 2014;128: 649–652. 10.5858/2004-128-649-EOCPPI 15163235

[18] Motta VP, Malafaia O, Ribas-Filho JM, Czeczko NG, Ribas CAPM, Cuenca RM. CASPASE-3 and CD-34 expression in prostate adenocarcinoma. Rev Col Bras Cir. 2009;36: 223–229. 20076902 10.1590/s0100-69912009000300008

[19] Campos EBP, Yoshida WB. The role of free radicals in the pathophysiology of ischemia and reperfusion in skin flaps: experimental models and treatment strategies. J Vas Bras. 2004;3: 357–366.

[20] Francisco Neto A, Silva JCCB, Fagundes DJ, Pericário S, Novo NF, Juliano Y, et al Oxidative alterations, total antioxidant status and nitric oxide study in rats submitted to ischemia and reperfusion of hind limbs. Acta Cir Bras. 2005;20: 134–139. 15884713 10.1590/s0102-86502005000200006

[21] Teixeira ARF, Molan NT, Kubrusly MS, Bellodi-Privato M, Coelho AM, Leite KR et al Post-conditioning ameliorates lipid peroxidation in liver ischemia-reperfusion injury in rats. Acta Cir Bras. 2009;24: 52–56. 19169543 10.1590/s0102-86502009000100011

[22] Freitas SH, Evencio Neto J, Doria RGS, Mendonca FS, Simões MJ, Camargo LM, et al Morphologic, morphometric, and ultrastructural aspects of the spleen of rats after total hepatic pedicle clamping. Arq Bras Med Vet Zootec. 2009;61: 1314–1321.

[23] Peralta C, Bulbena O, Xaus C, Prats N, Cutrin JC, Poli G, et al Ischemic preconditioning: a defense mechanism against the reactive oxygen species generated after hepatic ischemia reperfusion. Transplantation. 2002;73: 1203–1211. 11981410 10.1097/00007890-200204270-00004

[24] Fernandez L, Heredia N, Peralta C, Xaus C, Rosello-Catafau J, Rimola A, et al Role of ischemic preconditioning and the portosystemic shunt in the prevention of liver and lung damage after rat liver transplantation. Transplantation. 2003;76: 282–289. 10.1097/01.TP.0000067529.82245.4E 12883180

[25] Glantzounis GK, Salacinski HJ, Yang W, Davidson BR, Seifalian AM. The contemporary role of antioxidant therapy in attenuating liver ischemia-reperfusioninjury: A Review. Liver Transpl. 2005;11: 1031–1047. 10.1002/lt.20504 16123965

[26] Inouyea LA, Fernandeza LM, Carneiroa LFS, Germanoa JJ, Criscib AR. Morphological evaluation of liver and lung after poisoning by organophosphate in wistar rats. Uniciências. 2014; 18103–18109.

[27] Frederiks WM, James J, Bosch KS, Schroder MJR, Schuyt HCA. Model for provoking ischemic necrosis in rat liver. Parenchyma and its quantitative analysis. Exp Pathol. 1982;22: 245–252. 7160451 10.1016/s0232-1513(82)80015-7

[28] Asakava H, Jeppson B, Mack P, Hultberg B, Hagertrand I, et al Acute ischemic liver failure in the rat: a reproducible model not requiring portal decompression. Eur Surg Res. 1989;21: 42–48. 2714309 10.1159/000129002

[29] Isozaki H, Okajima K, Kobayashi M, Hara H, Akimoto H. Experimental study of liver injury after partial hepatectomy with intermittent or continuous hepatic vascular oclusion. Eur Surg Res. 1995;217: 313–322. 10.1159/000129415 7589003

[30] Gonce ME, Brackett DJ, Squires RA, Gibson DD, Kajdacsy-Balla A, Lerner LM, et al A new model for inducing total hepatic ischemia while preventing circulatory collapse. Secondary to splanchic vascularcongestion. Shock. 1995;3: 440–446. 7656069

[31] Hardy KJ, Tancheroen S, Shulkes A. Comparison of continuous versus intermittent: reperfusion during liver resection in an experimental model, J Surg. 1995;82: 833–836. 10.1002/bjs.1800820636 7627525

[32] Maruyama H, Harada A, Hurokawa T, Kobayashi H, Nonami T, Nakao A, et al Duration of liver ischemia and hepatic regeneration after hepatectomy in rats. J Surg Res. 1995;58: 290–294. 10.1006/jsre.1995.1045 7885025

[33] Chang L, Yu MC, Yuan YF, Shen WL, Wang HT, Liu QY, et al Safety and effectiveness of hemihepatic blood flow occlusion versus Pringle's maneuver during hepatectomy: a meta-analysis. CJEBM. 2014;14: 743–751. 10.1016/j.asjsur.2021.06.023 34246536

[34] Massip-Salcedo M, Roselló-Catafau J, Prieto J, Avila MA, Peralta C. The response of the hepatocyte to ischemia. Liver Int. 2007;27: 6–16. 10.1111/j.1478-3231.2006.01390.x 17241376

[35] Ramalho FS, Fernandez-Monteiro I, Rosello-Catafau J, Peralta C. Hepatic microcirculatory failure. Acta Cir Bras. 2006;21: 48–53. 17013514 10.1590/s0102-86502006000700012

[36] Quireze C Jr, Montero EFS, Leitão RM, Juliano Y, Fagundes DJ, Poli-de Figueiredo LF. Ischemic preconditioning prevents apoptotic cell death and necrosis in early and intermediate phases of liver ischemia-reperfusion injury in rats. J Invest Surg. 2006;19: 229–236. 10.1080/08941930600778206 16835137

[37] Montero EF, Quireze C Jr, d’Oliveira DM. Bile duct exclusion from selective vascular inflow occlusion in rat liver: role of ischemic preconditioning and N-acetylcysteine on hepatic reperfusion injury. Transplant Proc. 2005;37: 425–427. 10.1016/j.transproceed.2004.12.194 15808665

[38] Eum HA, Cha YN, Lee SM. Necrosis and apoptosis: sequence of liver damage following reperfusion after 60 min ischemia in rats. Biochem Biophys Res Commun. 2007;358: 500–505. 10.1016/j.bbrc.2007.04.153 17490613

[39] Kohli V, Selzner M, Madden JF, Bentley RC, Clavien PA. Endothelial cell and hepatocyte deaths occur by apoptosis after ischemia-reperfusion injury in the rat liver. Transplantation. 1999;67: 1099–1105. 10232558 10.1097/00007890-199904270-00003

